# Intrathymic Notch3 and CXCR4 combinatorial interplay facilitates T-cell leukemia propagation

**DOI:** 10.1038/s41388-018-0401-2

**Published:** 2018-07-23

**Authors:** Francesca Ferrandino, Giovanni Bernardini, Georgia Tsaouli, Paola Grazioli, Antonio Francesco Campese, Claudia Noce, Ambra Ciuffetta, Alessandra Vacca, Zein Mersini Besharat, Diana Bellavia, Isabella Screpanti, Maria Pia Felli

**Affiliations:** 1 Department of Experimental Medicine, Sapienza University, Viale Regina Elena 324, 00161 Roma, Italy; 2 Department of Molecular Medicine, Sapienza University, Viale Regina Elena 291, 00161 Roma, Italy; 3 IRCCS Neuromed, Via Atinense 18, 86077 Pozzilli, IS Italy

## Abstract

Notch hyperactivation dominates T-cell acute lymphoblastic leukemia development, but the mechanisms underlying “pre-leukemic” cell dissemination are still unclear. Here we describe how deregulated Notch3 signaling enhances CXCR4 cell-surface expression and migratory ability of CD4^+^CD8^+^ thymocytes, possibly contributing to “pre-leukemic” cell propagation, early in disease progression. In transgenic mice overexpressing the constitutively active Notch3 intracellular domain, we detect the progressive increase in circulating blood and bone marrow of CD4^+^CD8^+^ cells, characterized by high and combined surface expression of Notch3 and CXCR4. We report for the first time that transplantation of such CD4^+^CD8^+^ cells reveals their competence in infiltrating spleen and bone marrow of immunocompromised recipient mice. We also show that CXCR4 surface expression is central to the migratory ability of CD4^+^CD8^+^ cells and such an expression is regulated by Notch3 through β-arrestin in human leukemia cells. De novo, we propose that hyperactive Notch3 signaling by boosting CXCR4-dependent migration promotes anomalous egression of CD4^+^CD8^+^ cells from the thymus in early leukemia stages. In fact, in vivo CXCR4 antagonism prevents bone marrow colonization by such CD4^+^CD8^+^ cells in young Notch3 transgenic mice. Therefore, our data suggest that combined therapies precociously counteracting intrathymic Notch3/CXCR4 crosstalk may prevent dissemination of “pre-leukemic” CD4^+^CD8^+^ cells, by a “thymus-autonomous” mechanism.

## Introduction

Malignant transformation of T-cell progenitors is causative of T-cell acute lymphoblastic leukemia (T-ALL). T-ALL accounts for 15% of pediatric and 25% of adult ALL cases, very frequently bearing somatic gain-of-function gene mutations in Notch1, as well as overexpression of Notch3 [1–3]. Moreover, Notch3 gene activating mutations have been recently reported in T-ALL [4]. Notch receptors regulate T-cell fate choices, dominating early steps of thymocyte differentiation [5, 6]. Additionally, thymocyte turnover is regulated by natural cell competition, between “young” bone marrow (BM)-derived and “old” thymus-resident progenitors, whose impairment enables T-ALL progression via pre-malignant stages [7]. A major role is also played by the interaction between leukemia and non-leukemia cells in the microenvironment, probably dictating the survival of leukemia initiating cells.

Chemokines drive T-cell development through a gradient-dependent directional migration. Secreted by stromal and epithelial cells, chemokines mediate physiological and pathological processes, essentially related to cell homing and migration [8]. In adult thymus, T-cell precursors development requires CXCL12, also termed stromal derived factor-1 (SDF-1), which by binding to the G protein coupled receptor (GPCR), CXCR4, and through multiple divergent pathways, leads to chemotaxis, survival, and proliferation [8]. Through the cortex and medulla, GPCRs guide immature thymocytes to the appropriate microenvironment for specific developmental stages: CD4^−^CD8^−^ Double Negative (DN)1–4 to CD4^+^CD8^+^ Double Positive (DP) stages and CD4^+^ or CD8^+^ Single Positive (SP), respectively [9]. Moreover, SDF-1/CXCR4 axis is linked to mature SP thymocytes egress from the thymus [10, 11]. CXCR4, highly expressed since DN2 to the DP stage [12, 13], drives normal intrathymic T-cell development [14]. During β-selection, the SDF-1/CXCR4 axis cooperates with preTCR to allow Notch-dependent differentiation of DN3 to DP thymocytes. Moreover, CXCR4 regulates preTCR-dependent survival signals and maturation of thymocytes during β-selection [15]. This early selection is under the control of two Notch receptors, Notch1 mainly driving DN2 to DN3, while Notch3 governing DN3 to DP thymocyte transitions [16, 6]. Both preTCR and CXCR4 signals converge on Erk phosphorylation, regulating SDF-1-induced chemotaxis of DN3 thymocytes [17, 14].

We previously demonstrated the oncogenic potential of Notch3 in transgenic (tg) mice, overexpressing the constitutively active intracellular domain of Notch3 (N3-IC) in immature thymocytes, which develop an aggressive T-cell ALL, recapitulating most of human T-ALL features. Four-week-old N3-ICtg mice display early precursor deregulation, by expanding the DN3 stage and increasing total thymic cellularity [18]. At 12 weeks, thymus depletion, splenomegaly, lymph nodes enlargement, and BM colonization by lymphoblastic cell population occur. Phenotypic similarities between the infiltrating lymphoma cells and the thymocytes of younger mice suggested an immature T-cell propagation [18]. Notably, a prominent feature in Notch-induced T-ALL mouse models is the circulation of CD4^+^CD8^+^ T-cells [19, 20]. Moreover, disrupted natural cell competition in the thymus may enable progression to leukemia by dissemination of pre-T-ALL CD4^lo/+^/CD8^+^ cells [7].

Here, we study anomalous CD4^+^CD8^+^ T-cells propagation in Notch3-IC-induced T-ALL, by detecting atypical DP T-cells outside the thymus at early and late T-ALL stages. Notably, our results highlight that the high and combined expression of CXCR4 and Notch3 defines “pre-leukemic” DP-cells, precociously detected inside the thymus, and then in circulating blood and BM. Newly, by experiments of in vivo cell-transfer, we delineate the biological properties of CD4^+^CD8^+^Notch3^+^CXCR4^+^ thymocytes that are fit to infiltrate peripheral organs. Notably, in young transgenic N3-ICtg mice, the in vivo administration of the CXCR4 antagonist, AMD3100, can drastically reduce the infiltration of CD4^+^CD8^+^Notch3^+^CXCR4^+^ T-cells into BM. Interestingly, by ex vivo and in vitro experiments, we demonstrate that Notch3 modulates CXCR4 cell-surface expression through a β-arrestin-mediated mechanism, both in N3-ICtg mice-derived cells and in the human TALL-1 cell line, known to harbor Notch3 activating mutations [21].

Overall, our data suggest that in Notch3-induced T-ALL leukemia, high Notch3 and CXCR4 co-expression marks “pre-leukemic” DP thymocytes and allows their egression and propagation outside the thymus. This finally gives them access to the blood stream and BM and favors T-ALL progression.

## Results

### High CXCR4 cell-surface expression correlates with enhanced migration of N3-ICtg DP thymocytes

Aberrant preT/T-cell development, Protein Kinase C θ-dependent constitutive activation of NF-κB, persistent expression of pTα/preTCR chain, and a failure of CD25 downmodulation beyond the DP stage characterize N3-ICtg mice [18, 2, 22, 23].

SDF-1/CXCR4 axis-mediated DN/DP transition and its suggested relevance in T-ALLs [14] prompted us to analyze CXCR4 expression in abnormally represented DP T-cells in N3-ICtg mice.

**Fig. 1:**
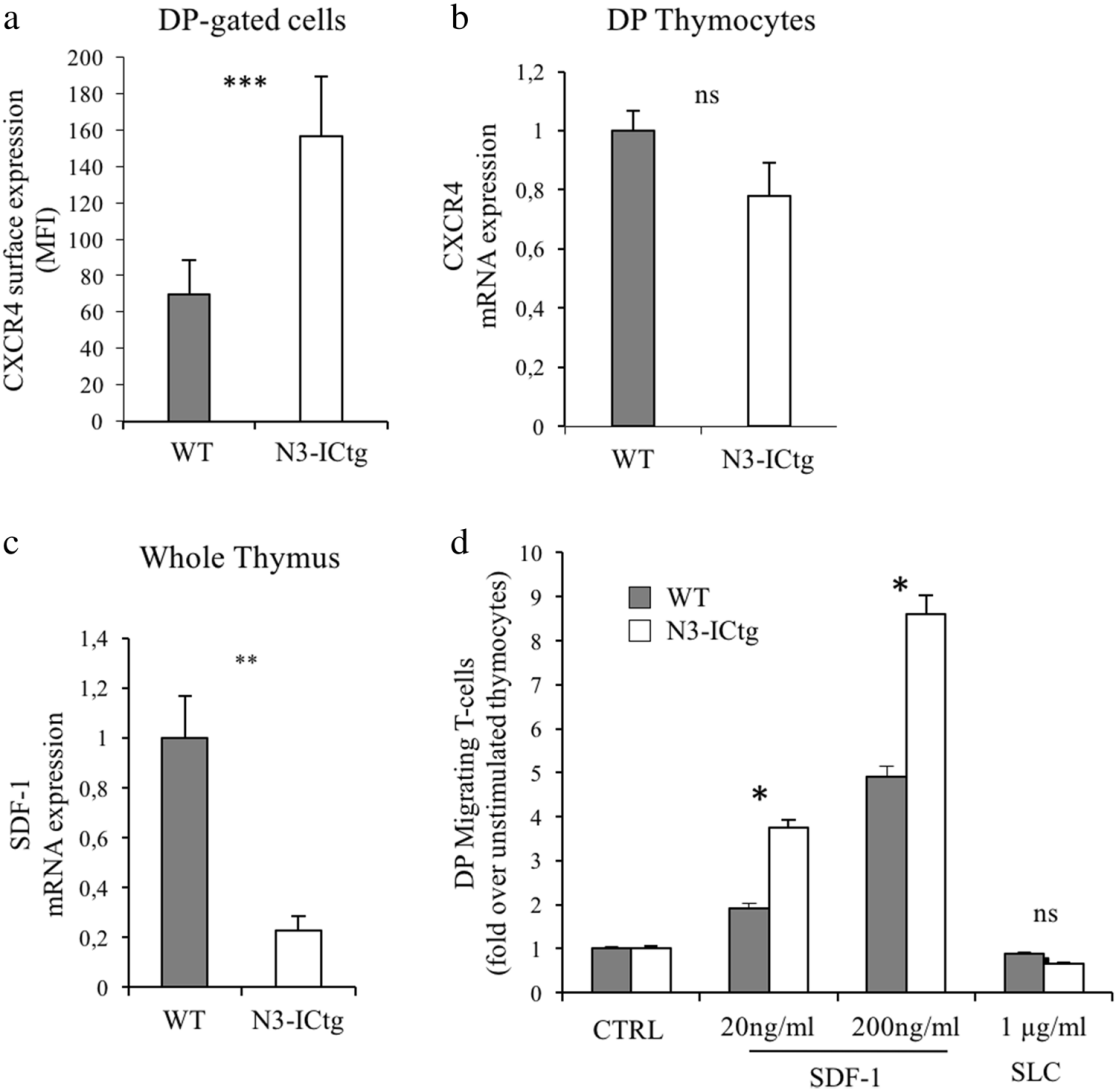
Enhanced CXCR4 cell-surface expression and migratory ability in N3-ICtg thymocytes. **a** surface CXCR4 mean fluorescence intensity (MFI) on DP-gated thymocytes of WT (*n* = 4) and N3-ICtg (*n* = 5) mice; **b** CXCR4 mRNA expression in DP thymocytes of WT (*n* = 3) and N3-ICtg (*n* = 3) mice; **c** SDF-1 mRNA expression in whole thymi of WT (*n* = 3) and N3-ICtg (*n* = 3) mice; **d** Migration of WT (*n* = 3) and N3-ICtg (*n* = 3) DP thymocytes in response to (20–200 ng/ml) SDF-1, evaluated as fold increase of DP migrating thymocytes percentages in SDF-1 stimulated versus unstimulated thymocytes (CTRL). Unrelated ligand, SLC. Each experiment was performed with 6–8-week-old mice. Results represent mean ± SD. (*p < 0.05; **p<0.01; ***p < 0.001; ns, not significant; Student’s *t*-test)

First, we observed a higher CXCR4 cell-surface expression in transgenic DP thymocytes at the single cell level (Fig. 1a), as highlighted by the significant difference in the CXCR4 mean fluorescence intensity (MFI) between wild-type (WT) and N3-ICtg DP-gated thymocytes of 6–8-week-old mice. Increased CXCR4 expression seems to not be transcriptionally regulated (Fig. 1b). Moreover, in DP thymocytes of N3-ICtg as well as in WT mice, SDF-1 mRNA is undetectable (data not shown). Our data propose a leukemic cell-autonomous mechanism, excluding SDF-1 autocrine production, as previously reported [24, 25]. In addition, we observed a greatly decreased expression of SDF-1 mRNA in N3-ICtg as opposed to WT whole thymus (Fig. 1c).

SDF-1/CXCR4 axis-mediated chemotaxis regulates thymocyte maturation by directing DP T-cells into the cortex [26] and retaining them there. In our model, enhanced cell-surface expression correlates to CXCR4 function. In fact, in response to SDF-1, N3-ICtg DP thymocyte migration increases more than WT DP cell migration and does so in a dose-dependent manner (Fig. 1d).

Conceivably, transgenic DP thymocytes, with higher CXCR4 surface expression, are more responsive to SDF-1, whose reduced expression in the N3-ICtg thymus microenvironment (Fig. 1c) and decreased retaining function may deregulate their progressive maturation into the thymic cortex and increase their responsivity to external stimuli.

### Notch3^+high^CXCR4^+high^DP T-cells competitively increase in N3-ICtg thymus

**Fig. 2:**
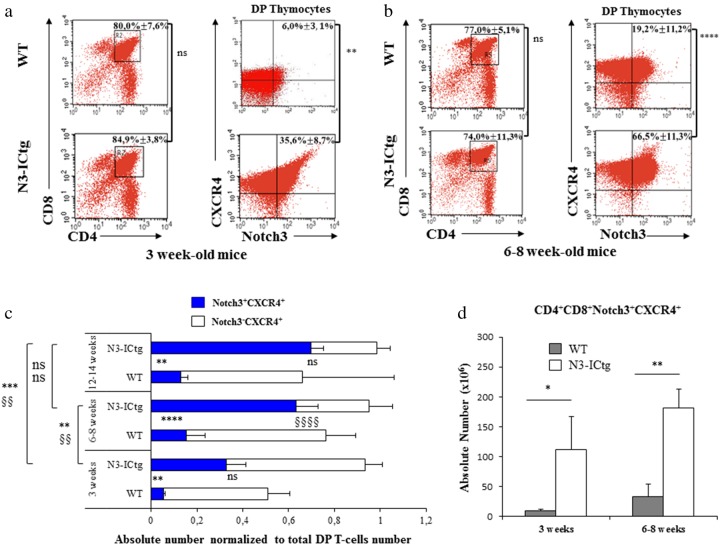
High Notch3^+^CXCR4^+^ cell-surface co-expression characterizes N3-ICtg DP thymocytes. **a** Left: thymocytes subset distribution with average DP percentage ± SD. Right: DP-gated thymocytes with average percentage ± SD of Notch3^+^CXCR4^+^DP cells. WT (*n* = 3) and N3-ICtg (*n* = 4) mice. **b** Left: thymocytes subset distribution with average DP percentage ± SD; right: DP-gated thymocytes with average percentage ± SD of Notch3^+^CXCR4^+^DP cells. WT (*n* = 7) and N3-ICtg (*n* = 9) mice. **c** Absolute numbers of Notch3^+^CXCR4^+^ and/or Notch3^-^CXCR4^+^DP normalized to total DP thymocytes values. Student’s *t*-test between WT and N3-ICtg at each age; symbols § and * indicate Notch3^−^CXCR4^+^ and Notch3^+^CXCR4^+^, respectively. For each corresponding age, WT (*n* = 4) and N3-ICtg (*n* = 5) mice were used. One-way ANOVA and Tukey’s post-test (left side) among different aged N3-ICtg (ns, not significant, **p < 0.01; ***p<0.001; ****p < 0.0001; ^§§§§^*p* < 0.0001; ^§§^*p* < 0.01). **d** CD4^+^CD8^+^Notch3^+high^CXCR4^+high^ absolute numbers in N3-ICtg (*n* = 3) versus WT (*n* = 3) mice (*p < 0.05; **p < 0.01). See also Figure S1

Relative to WT, DP T-cell percentages of 3– and 6–8-week-old transgenic thymi are unchanged (Fig. 2a, b), while slightly decreased in 12–14-week-old mice (Figure S1A). High CXCR4 (Fig. 1a) and the persistent Notch3 cell-surface expression (Figure S1B), prompted us to evaluate Notch3 and CXCR4 combined expression in CD4^+^CD8^+^ thymocytes at different ages.

In 3-week-old thymi, DP-gated thymocytes display a higher percentage of Notch3^+^CXCR4^+^ cells in N3-ICtg versus WT mice (35.6% ± 8.7% versus 6.0% ± 3.1%) (Fig. 2a, right panels), thus indicating their increase before full-blown disease. CD4^+^CD8^+^Notch3^+^CXCR4^+^ thymocytes of 6–8-week-old N3-ICtg mice strongly increase (66.5% ± 11.3%) within the DP population and stain more brightly (Fig. 2b). Notably, the very few Notch3^+^CXCR4^-^DP thymocytes in N3-ICtg mice suggest that the Notch3 signal forces DP thymocytes to become Notch3^**+**high^CXCR4^**+**high^. This is sustained by the strong increase in their absolute numbers in N3-ICtg as opposed to in WT mice (Fig. 2d). We then compared the absolute numbers of different subsets of DP cells, by normalizing the number of DP cells bearing only one or both the receptors (CXCR4 and/or Notch3) to DP thymocyte total number. Respectively, Notch3^−^CXCR4^+^ or Notch3^+^CXCR4^+^ versus total DP thymocytes, at different ages, from younger (3-week old) to older mice (12–14-week old) have been analyzed. With respect to WT, 3-week-old transgenic thymus displays a significant expansion of CD4^+^CD8^+^Notch3^+^CXCR4^+^ thymocytes, further increasing in 6–8 and persisting in 12–14-week-old mice (Fig. 2c). At each age, N3-ICtg thymus is progressively more populated by CD4^+^CD8^+^Notch3^+high^CXCR4^+high^, when compared to WT thymus. Conversely, CD4^+^CD8^+^Notch3^−^CXCR4^+high^ T-cell number decreases with age in N3-ICtg thymus (Fig. 2c).

Clearly, hyperactive Notch3 induces an overcoming DP subset, which brightly expresses Notch3 and CXCR4 receptors.

### Anomalous Notch3^+^CXCR4^+^DP T-cells are detected early in circulating blood of N3-ICtg

Infiltration of CD4^+^CD8^+^ T-cells in peripheral lymphoid organs has already been demonstrated in preclinical models of Notch-induced T-ALL [18, 27, 20]. Moreover, Pin1 deletion prevents leukemia progression by reducing Notch3 expression and blocking the expansion/invasiveness of circulating CD4^+^CD8^+^ T-cells in N3-ICtg blood [21].

**Fig. 3:**
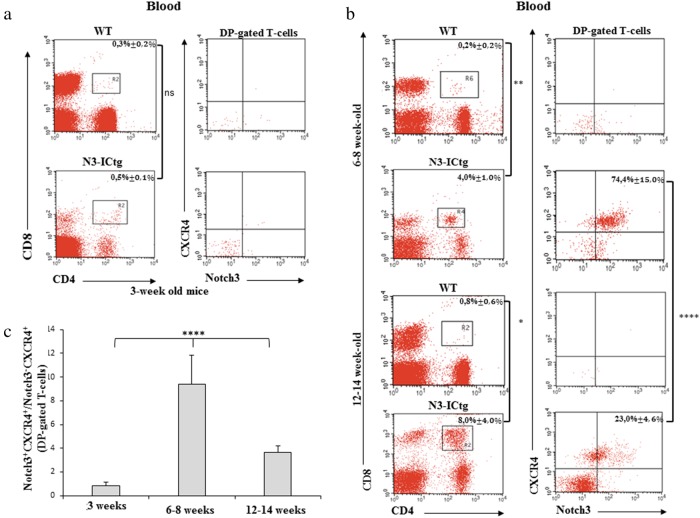
High Notch3^+^CXCR4^+^DP T-cells in circulating blood of N3-ICtg mice. **a** CD4/CD8 flow-cytometry analysis of blood from WT (*n* = 3) and N3-ICtg (*n* = 3) mice (3-week old). **b** Left: representative plots of CD4^+^CD8^+^ circulating in 6–8 and 12–14-week-old WT (*n* = 4) and N3-ICtg (*n* = 4) blood; Right: Notch3^+^CXCR4^+^ within DP-gated T-cells. The average percentages ± SD of three to four analyzed animals are indicated. **c** Ratio of the percentages of Notch3^+^CXCR4^+^ as fold over Notch3^−^CXCR4^+^ in DP-gated cells in N3-ICtg mice. For each corresponding age, at least four N3-ICtg mice were analyzed. (*p<0.05; **p<0.01; ****p < 0.0001; ns, not significant; One-way ANOVA-test). See also Figure S2

Given the competitive increase of Notch3^+high^CXCR4^+high^DP thymocytes in 3-week-old N3-ICtg mice (Fig. 2a) and CXCR4-mediated SP cell egress, we evaluated the immunophenotype of atypical CD4^+^CD8^+^ cells in circulating blood (Fig. 3). Before the full-blown disease, the blood of 3-week-old N3-ICtg mice is virtually devoid of DP T-cells (0.5% ± 0,1%), as compared to WT mice (0.3% ± 0.2%) (Fig. 3a, left panels). Consequently, Notch3^+^CXCR4^+^DP T-cells are virtually undetectable (Fig. 3a, right panels). Conversely, by 6–8 weeks (Fig. 3b, upper panels), we find CD4^+^CD8^+^ (4.0% ± 1.0%) in N3-ICtg blood, mostly composed of Notch3^+high^CXCR4^+high^DP cells (74,4% ± 15,0%), recalling the specific Notch3^+high^CXCR4^+high^DP transgenic thymocytes (Fig. 2b). At 12–14 weeks, when the disease progresses (Fig. 3b, lower panels), DP T-cells percentage increases in N3-ICtg blood (8,0% ± 4,0%). Within this population of DP T-cells, Notch3^+^CXCR4^+^ (23,0% ± 4,6%) are progressively replaced by Notch3^−/low^CXCR4^−^ T-cells. Consequently, CD4^+^CD8^+^Notch3^+high^CXCR4^+high^ cells portray the early circulating DP T-cells in the blood of our T-ALL model.

In support of deregulated CXCR4 expression, transgenic CD8^+^ thymocytes show a decreased percentage of CXCR4^+^ cells and a reduced SDF-1-induced migration when compared to CD8^+^ WT thymocytes (Figure S2A and B). It is possible that Notch3 activation impairs thymocyte development beyond the DP stage by favouring precocious exit of DP thymocytes from the thymus, finally affecting CD8^+^ T-cells maturation.

Based on the ratio between CD4^+^CD8^+^Notch3^+^CXCR4^+^ and CD4^+^CD8^+^Notch3^-^CXCR4^+^ T-cell percentage, we observed in 6–8-week-old N3-ICtg blood stream the maximum expansion of circulating Notch3^+^CXCR4^+^DP T-cells (Fig. 3c), which overcome Notch3^−^CXCR4^+^DP T-cells. At 12–14 weeks, with the disease fully developed, the ratio of Notch3^+^CXCR4^+^ versus Notch3^-^CXCR4^+^ DP subsets within the total DP population is greatly decreased (Fig. 3c), as the data in Fig. 3b (lower panels) show.

Our results suggest a dynamic trend, with a time-dependent switch of circulating DP T-cell subsets in N3-ICtg blood. Propagation of Notch3^+high^CXCR4^+high^DP T-cells outside the thymus may be a result of the abnormal increase within young N3-ICtg thymus (Fig. 2a) and their enhanced SDF-1-dependent migratory ability (Fig. 1d), possibly related to the decreased expression of thymic SDF-1 (Fig. 1c), which may justify the decreased retention inside the thymus.

### The wave of Notch3^+^CXCR4^+^DP T-cells progressively invades N3-ICtg BM

CXCR4 expression facilitates pathogenic T-cell trafficking in the BM of murine Aplastic Anemia model [28]. Moreover, BM stroma producing SDF-1 and vascular endothelial niche are necessary for leukemic cell maintenance in T-ALL progression [25].

**Fig. 4:**
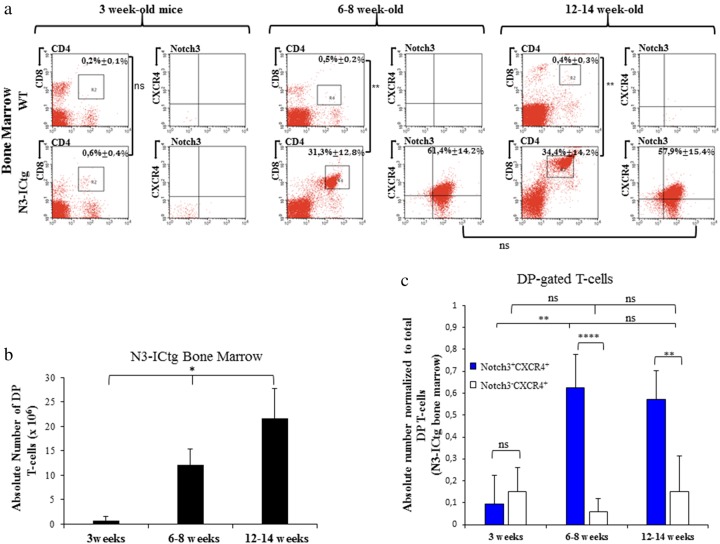
Notch3^+^CXCR4^+^DP T-cells increasingly infiltrate N3-ICtg BM with disease progression. In **a**, 3 weeks, 6–8 weeks, and 12–14 weeks of age WT (*n* = 4) and N3-ICtg (*n* = 6) BM. On the left of each figure section: representative plots of T-cell subset distribution. On the right: Notch3^+^CXCR4^+^ co-expression within DP-gated T-cells. The average percentages ± SD are shown. (**p<0.01; ns, not significant). **b** Absolute numbers of DP cells infiltrating transgenic (*n* = 3) BM (*p < 0.05, one-way ANOVA test). **c** Absolute numbers of Notch3^+^CXCR4^+^ and/or Notch3^−^CXCR4^+^DP-cells normalized to total DP gated cells of N3-ICtg bone marrow. For each corresponding age, at least five N3-ICtg mice were used. One-way ANOVA (Tukey’s post-test) and Student’s *t*-test performed where appropriate (**p < 0.01; ****p < 0.0001; ns, not significant)

To analyze BM infiltration in our mouse model, we first assessed the percentages of DP T-cells in BM of 3-week-old WT (0.2% ± 0.1%) and N3-ICtg (0.6% ± 0.4%) mice (Fig. 4a). Essentially, Notch3^+^CXCR4^+^ DP T-cells were undetectable.

With disease progression, a massive infiltration of N3-ICtg BM was observed (Fig. 4a). Indeed, we assessed the higher percentage of total DP T-cells in N3-ICtg BM at 6–8 weeks (31.3% ± 12.8%), and at 12–14 weeks of age (34.4% ± 14.2%), as opposed to 3-week-old mice (0.6% ± 0.4%) (Fig. 4a). In contrast, as shown in Fig. 4a, DP cells were never detected in WT BM at 6–8 and 12–14 weeks of age (0.5% ± 0.2% and 0.4% ± 0.3%, respectively). Meanwhile, at different ages, a statistically significant increase of the absolute number of total DP T-cells infiltrating N3-ICtg BM was observed (Fig. 4b).

Notch3^+^CXCR4^+^ T-cells mostly compose the DP population infiltrating the BM in 6–8-week old (61.4% ± 14.2%), with a steady level in 12–14-week-old (57.9% ± 15.4%) N3-ICtg mice (Fig. 4a). Given these high percentages, we speculate that this population colonizes and easily proliferates in the transgenic BM niche. Thus, we compared the absolute numbers of Notch3^−^CXCR4^+^DP versus Notch3^+^CXCR4^+^ DP cells, normalized to total DP T-cell values (Fig. 4c). No difference between the two subsets in 3-week-old mice was observed, in agreement with data shown in Fig. 3c. Conversely, Notch3^+^CXCR4^+^DP cell number peaks at 6–8 weeks and remains unchanged in 12–14-week-old N3-ICtg mice (Fig. 4c).

Newly, our results highlight the homing and expansion of Notch3^+^CXCR4^+^ DP-cells in N3-ICtg BM, during T-ALL progression.

The association of CXCR4 expression to site-specific metastasis, into BM [29, 30] of neuroblastoma-bearing patients [31], and the supportive effect of endothelial niche in Notch-dependent T-ALL cells maintenance [25, 24], favor our hypothesis that Notch3/CXCR4 crosstalk directs “pre-leukemic” DP-cells to the BM.

### High Notch3/CXCR4 co-expression facilitates DP thymocytes homing and engraftment into BM

The “bone marrow-derived” leukemic cells, CXCR4-dependent invaders [25, 24], T lymphoid precursors clonal proliferation, and the suggested intrathymic origin of T-ALL [32, 33] prompted us to analyze whether the Notch3^+^CXCR4^+^DP thymocytes, with high capability to migrate and circulate in N3-ICtg mice, could specifically engraft and infiltrate into BM of NSG recipient mice. In Figure S3A, our separation protocol is schematized.

**Fig. 5:**
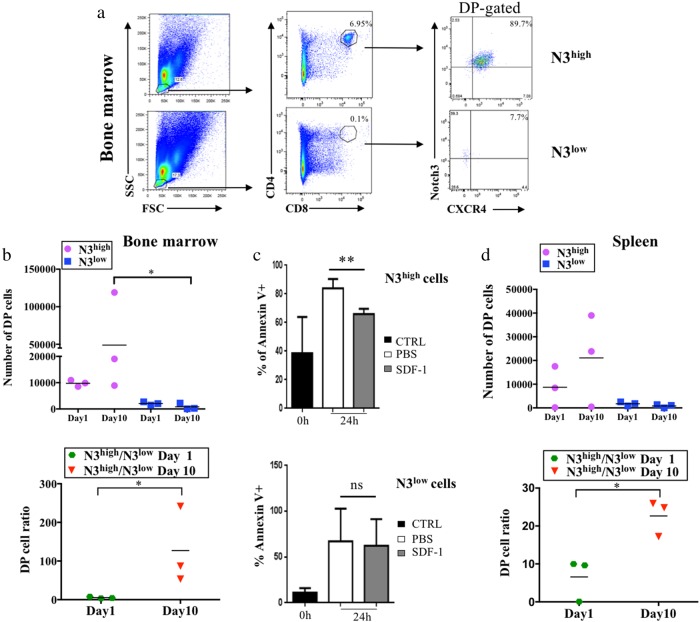
Selective homing and engraftment of N3-ICtg DP thymocytes, highly expressing Notch3 and CXCR4. **a** (CD4^+^CD8^+^Notch3^+high^CXCR4^+high^) N3^high^ or (CD4^+^CD8^+^Notch3^+low^CXCR4^+high^) N3^low^ thymocytes engraft in BM day10 post-intravenous injection (i.v.). Percentages of DP cells (middle) and of CD4^+^CD8^+^Notch3^+^CXCR4^+^ (right). **b** Absolute numbers of DP infiltrating NSG BM after N3^high^ (purple-circle) or N3^low^ (blue-square) thymocytes i.v. (upper, Kruskal–Wallis test) and the ratio N3^high^/N3^low^DP cells at day1 (green-hexagon) and day10 (red-triangle) (lower, Mann–Whitney test) (*p<0.05). **c** Ex vivo experiments: percentages of AnnexinV^+^cells in N3^high^ (upper) and N3^low^ (lower) thymocytes in 24 h SDF-1-treated relative to saline (PBS). Data are represented as meanvalue ± SD (**p < 0.01; ns, not significant; Student’s *t*-test). **d** Absolute DP T-cell numbers in spleen after N3^high^ (purple-circle) or N3^low^ (blue-square) DP thymocytes i.v. (upper) and the ratio of N3^high^/N3^low^DP T-cells (lower) at day1 (green-hexagon) and day10 (red-triangle) (*p<0.05; Student’s *t*-test, Mann–Whitney). See also Figure S3A and B

Purified CD4^+^CD8^+^ thymocytes of 6–8-week-old transgenic mice, immunophenotypically distinguished in Notch3^+high^CXCR4^+high^ (N3^high^) or Notch3^+low^CXCR4^+high^ (N3^low^) (Figure S3B) were injected intravenously (i.v.) into NSG recipient mice. At day10 post-injection, BM cells from mice receiving N3^high^ cells, show a high percentage of donor DP T-cells (6.95%), mostly composed of CD4^+^CD8^+^Notch3^+high^CXCR4^+high^ cells (89.7%) (Fig. 5a, upper panels). Conversely, BM cells from N3^low^-injected mice display only 0.1% of DP T-cells, essentially devoid of surface Notch3 (Fig. 5a, lower panels). The evaluation of DP T-cell numbers in BM of N3^high^-injected mice (Fig. 5b) evidences their trend to greatly increase, by comparing DP cell numbers at day1 and day10 (upper graph). Already at day1, DP T-cell number is higher in N3^high^-injected when compared to N3^low^-injected mice. In fact, at day1, N3^low^DP cells fail to home in NSG BM (Fig. 5b). Strikingly at day10, we demonstrate that N3^high^DP cells achieve a greatly increased engraftment in the BM of NSG recipient mice when compared to N3^low^DP cells (Fig. 5b). The difference is clearly indicated by the increased ratio of DP cell numbers (Fig. 5b, lower graph) from N3^high^-injected versus N3^low^-injected mice at day10. This evidence confirms the higher competence of N3^high^ thymocytes to home already at day1 and to engraft and expand in the BM of recipient mice 10 days post-injection. This “fitness” may rely on the enhanced responsivity of N3^high^ cells to BM microenvironment, releasing the pro-survival factor SDF-1. To this regard, in ex vivo experiments, 24 h after SDF-1 treatment, we observed a significant reduction of spontaneous apoptosis, as measured by AnnexinV staining (AnnnexinV^+^) within N3^high^DP thymocytes subset (Fig. 5c, upper panel), when compared to N3^low^DP cells, which appear quite SDF-1 unresponsive (Fig. 5c, lower panel). We hypothesize that Notch3 hyperexpression, through the increased surface expression of CXCR4 (Fig. 1a), allows a better DP settlement by potentiating SDF-1 responsiveness and hence CXCR4-mediated survival programs. Interestingly, N3^high^ and N3^low^DP cells have a different proliferation rate. In vivo BrdU labeling demonstrates that N3^high^DP cells have an enhanced proliferation rate (Figure S4A and B). Additionally, in comparison to N3^low^, N3^high^DP subset displays a higher Ki67 expression as confirmed by higher Ki67 MFI (Figure S4C).

Propagation of purified thymocytes was further analyzed in the spleen. By day1 post-injection, N3^high^DP-cells invade the spleen, further increasing at day10. In contrast, N3^low^ cells are nearly absent at both times (Fig. 5d, upper graph). Accordingly, the ratio of Notch3^+high^CXCR4^+high^ versus Notch3^+low^CXCR4^+high^ (N3^high^/N3^low^) DP cells increases, sustaining the competence of N3^high^ to be successful in infiltrating both BM and spleen (Fig. 5d, lower graph). Overall, we hypothesize that Notch3/CXCR4 crosstalk enhances the early spreading of N3^high^DP thymocytes toward BM and the spleen in response to SDF-1 chemoattraction, thus evidentiating their “fitness” in dissemination and expansion.

### CXCR4 in vivo pharmacological antagonism reduces BM infiltration by Notch3 overexpressing DP T-cells

AMD3100 is an extremely specific and effective CXCR4 antagonist. It has been used for hematopoietic stem cell mobilization as well as for the treatment of myeloid leukemia and solid tumors [34].

**Fig. 6:**
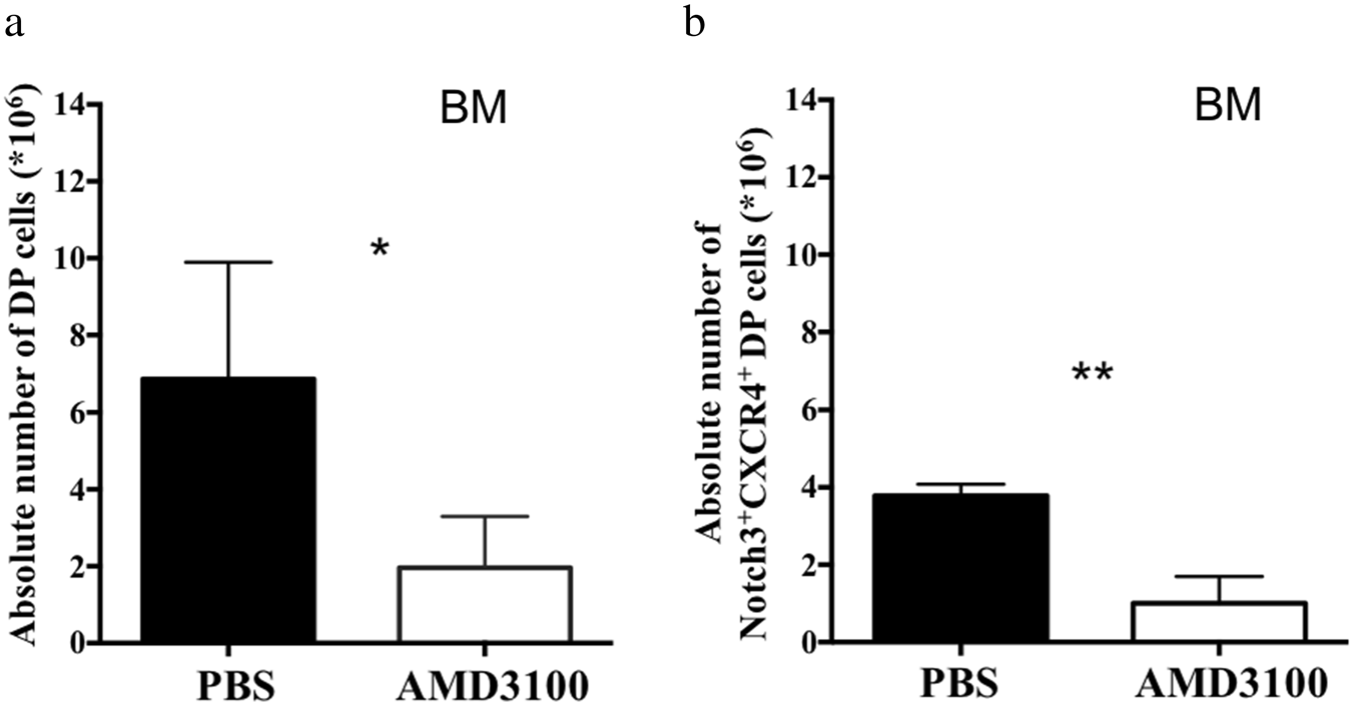
In vivo CXCR4 antagonism greatly contrast atypical DP infiltration in N3-ICtg BM. Three- to four-week-old N3-ICtg mice were treated daily with PBS (PBS) or with the CXCR4 antagonist, AMD3100. At day10 post-injection, absolute numbers **a** of DP T-cells and **b** of Notch3+CXCR4+DP T-cells were analyzed. PBS, *n* = 3; AMD3100, *n* = 6. Results represent mean ± SD (*p < 0.05; **p < 0.01; Student’s *t*-test)

To investigate the effect of CXCR4 antagonism in abnormal Notch3^+^CXCR4^+^DP cell propagation, we performed a daily in vivo intraperitoneal (IP) administration of young N3-ICtg mice, just before BM colonization occurs. After 10 days, we evaluated DP T-cell numbers in BM by flow cytometry, as shown in Fig. 6. Total DP cells that infiltrate BM are greatly reduced by the administration of AMD3100, as compared to PBS-injected mice (Fig. 6a). Interestingly, infiltrating DP cells are mostly composed by Notch3^+^CXCR4^+^, still significantly decreased in AMD3100-treated with respect to PBS-injected mice (Fig. 6b). Notably, these results demonstrate that in vivo CXCR4 pharmacological antagonism can dramatically reduce the BM infiltration and engraftment by “pre-leukemic” DP cells. Therefore, this in vivo treatment may have significant implications for a potential therapeutic approach.

### Notch3 targets CXCR4 surface expression by modulating β-arrestin1 function

**Fig. 7:**
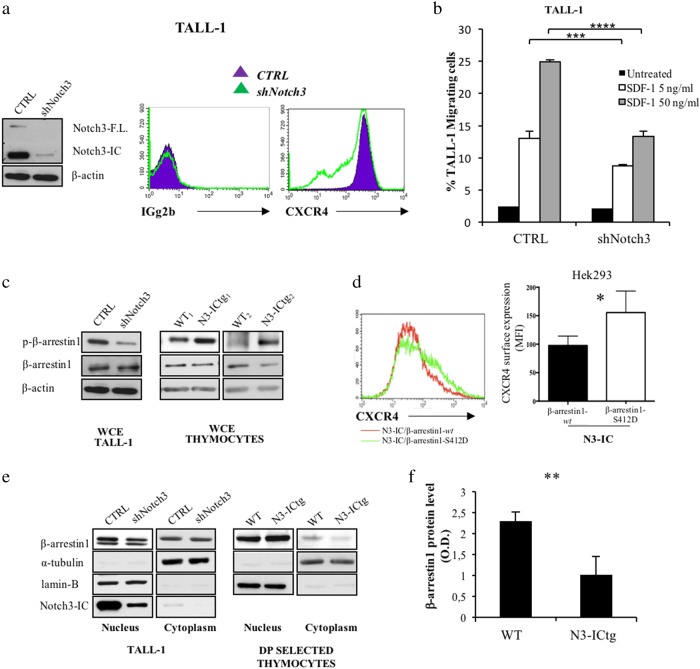
β-arrestin1-mediated CXCR4 cell-surface modulation by Notch3 in human leukemic cells. **a** Left: western blot analysis of Notch3 protein. Right: flow-cytometry of CXCR4 cell-surface expression in scrambled (CTRL) versus specific Notch3 silenced (shNotch3) TALL-1 cells. **b** Transwell migration assay of CTRL and shNotch3 cells in response to SDF-1 (5–50 ng/ml) (****p < 0.0001; ***p < 0.001, Student’s *t*-test). **c** Immunoblot assay of whole-cell extract (WCE) of CTRL and shNotch3 cells (left), and thymocytes from WT (WT_1_,_2_) and N3-ICtg (N3-ICtg_1_,_2_) mice (right). Phosphorylated (p-β-arrestin1) and unphosphorylated (β-arrestin1) β-arrestin1 protein levels. **d** Flow-cytometry analysis of CXCR4 expression of Hek293 transiently transfected with N3-IC (0.7 μg) either in the presence (1.4 μg) of β-arrestin1-*wt* (*wt*) or of the mutant S412D (β-arrestin1-S412D). Right panel CXCR4 MFI of at least five experiments (*p < 0.05, Student’s *t*-test). **e** Fractionated extract (Nucleus, Cytoplasm) of CTRL and shNotch3 TALL-1 cells and from DP/CD8^+^ thymocytes of WT and N3-ICtg 6–8-week-old mice. See also Figure S4 and S6. **f** Optical densitometry of β-arrestin1 protein in WT (*n* = 3) and N3-ICtg (*n* = 3) thymocytes (**p < 0.01, Student’s *t*-test). Densitometry was performed on scanned immunoblot images using ImageJ

For a molecular insight in Notch3-boosted CXCR4 expression, we modulated Notch3 function in a human leukemia cell-line, TALL-1 cells, characterized by CD4/CD8/CD3 expression, Notch3 activating mutations without known alterations of Notch1 [4], and here, for the first time, by high CXCR4 cell-surface expression (Fig. 7a).

We silenced the Notch3 gene in TALL-1 cells (shNotch3) and hence we monitored CXCR4 cell-surface expression by flow-cytometry, (Fig. 7a and S5). Notch3 silencing impairs surface expression of CXCR4 (Fig. 7a, right panel), which is not accounted for by reduced CXCR4 total protein levels (Figure S6A). In keeping with unchanged CXCR4 mRNA levels in N3-ICtg with respect to WT DP-cells (Fig. 1b), no Notch-induced transcriptional regulation of CXCR4 mRNA has been reported in T-ALL1 cells [35]. Notably, silencing of Notch3 decreases CXCR4 cell-surface expression, while greatly impairing TALL-1 in vitro migration in response to SDF-1 (Fig. 7b).

CXCR4 internalization [36, 37] implies β-arrestin1 activity, whose Erk-triggered inhibitory control regulates β-adrenergic receptor internalization [38]. Phosphorylation/dephosphorylation at Ser-412 selectively regulates endocytic but not desensitization function of β-arrestin [38]. With respect to control (CTRL), shNotch3 cells display a reduced expression level of Ser-412-phosphorylated β-arrestin1 (p-β-arrestin1), with respect to its unphosphorylated form (Fig. 7c, left panel). This is in agreement with decreased phospho/unphospho β-arrestin1 ratio (Figure S6B). Together, these results correlate with decreased phospho-Erk kinase in Notch3-silenced cells (Figure S6C), and are in agreement with the Erk signaling enhancement reported in DP/CD8^+^ thymocytes from N3-ICtg mice [39, 40]. In keeping with and supporting the above observations, the whole-cell protein extracts (WCE) of thymocytes from 6–8-week-old N3-ICtg mice (N3-ICtg_1_,_2_) display a higher level of p-β-arrestin1, when compared to thymocytes from WT mice (Fig. 7c, right panels).

To investigate the role of β-arrestin1 in mediating the Notch3 effect on CXCR4 expression, we utilized a β-arrestin1 mutant that mimics its phosphorylated form Ser412→Asp (S412D) and acts as a dominant negative inhibitor of receptor sequestration/internalization [41]. To this purpose, we transiently cotransfected Hek293 cells with the active Notch3-IC together with either the WT (β-arrestin1-*wt*) or the phosphorylated mutant (β-arrestin1-S412D) (Figure S7). As shown in Fig. 7d, we observed that, in the presence of activated Notch3, the constitutively phosphorylated form of β-arrestin1 (β-arrestin1-S412D) enhances CXCR4 surface expression more efficiently with respect to the *wt* form.

Together, the above results demonstrate that Notch3 is able to sustain the levels of p-β-arrestin1, which in turn cooperates with Notch3 to maintain the levels of CXCR4 surface expression.

Notably, Notch3 silencing (shNotch3) reduces β-arrestin1 in nuclear extract relative to CTRL cells (Fig. 7e, left panels). Indeed, DP/CD8^+^ thymocyte fractionated extracts reveal that β-arrestin1 protein increases in the nucleus, but decreases in the cytoplasm of N3-ICtg mice (Fig. 7e, right panels; and Figure S8A and B) supporting its inability to internalize CXCR4.

Newly, our results correlate Notch3 hyperexpression to altered β-arrestin1 function/localization, which impairs CXCR4 internalization.

Finally, since we observed a significant decrease of β-arrestin1 protein levels in thymocytes from N3-ICtg when compared to WT mice (Fig. 7f), which would further support its role in regulating CXCR4 expression, in order to investigate a possible translational significance of the correlation between the CXCR4 receptor and the β-arrestin1 adaptor/scaffold protein, we performed an in silico analysis of CXCR4 and β-arrestin1 gene expression in a cohort of 117 T-ALL pediatric patients [42]. Interestingly, we found a significant inverse correlation between CXCR4 and β-arrestin1 gene expression in selected patient subgroups (Figure S9A and B).

## Discussion

We characterized the impact of the relative “fitness” of thymus-derived “pre-leukemic” DP-cells on BM homing and engraftment. Still poorly understood are clues on what goes astray when in a normal thymus originates T-cell leukemia.

Gene expression profiling of human T-ALL samples, distinguishes different stages of arrest during T-cell development: (i) early immature T-ALLs at the DN stage, opposed to (ii) CD1a^+^/CD4^+^/CD8^+^ or CD3^+^/CD4^+^/CD8^+^ T-cells, respectively, in early-cortical and late-cortical thymocyte T-ALLs [43]. We report here the competitive progressive expansion of CD4^+^CD8^+^Notch3^+high^CXCR4^+high^ thymocytes in 3-week-old N3-ICtg thymus, peaking at 6–8 weeks and reaching a plateau at 12–14 weeks, well correlated to the anomalous propagation of DP-cells in circulating blood. Enhanced CXCR4 cell-surface expression characterizes highly migrating transgenic DP thymocytes and may interfere with normal T-cell/stroma interactions, thus relieving Notch3^+high^CXCR4^+high^DP cells from outside control. Our data converge on a “thymus-autonomous” mechanism also relying on reduced SDF-1-dependent retention inside the thymus of transgenic as opposed to WT intrathymic microenvironment. Hence, early detection of Notch3^+high^CXCR4^+high^DP in blood and BM of N3-ICtg mice at 6–8 weeks of age may suggest DP accelerated egress and thymus depletion, possibly unbalancing thymocyte sub-populations. As a possible consequence of this, T-cell depletion and subverted architecture in 12–14-week-old N3-ICtg thymus occur as previously reported [18]. Our results demonstrate the transient presence of Notch3^+high^CXCR4^+high^DP in circulating blood, like a wave of early propagating cells, further suggesting that Notch3^+high^CXCR4^+high^ expression is a possible “tag” to precociously detect abnormally circulating DP-cells. In contrast, 12–14-week-old N3-ICtg BM is still largely populated by Notch3^+high^CXCR4^+high^ DP-cells, suggesting that Notch3 makes them more fit to BM colonization. Indeed, SDF-1-producing vascular endothelial cells required in T-ALL niche [25] represents a supportive microenvironment, chemoattracting and favoring the expansion of Notch3^+high^CXCR4^+high^DP cells in BM vascular niches.

We demonstrate that Notch3 modulates surface expression of CXCR4 in human TALL-1, by requiring β-arrestin1, whose unphosphorylated status is necessary for β-arrestin/*src* and β-arrestin/clathrin interaction and GPCR/CXCR4 receptor endocytosis [44, 45]. Intriguingly, the β-arrestin1-S412D mutant, unable to bind clathrin and acting as a dominant negative inhibitor of receptor sequestration/internalization [41], enhances Notch3-induced CXCR4 cell-surface expression. We observed here that in N3-ICtg DP thymocytes, β-arrestin1 undergoes heavy Ser412-phosphorylation and increased nuclear translocation, suggesting the impairment of its function toward CXCR4 internalization. Additionally to previous data [24] linking CXCR4 recycling to calcineurin-dependent expression of cortactin, we propose that Notch3 regulates function and expression of β-arrestin1 to prevent CXCR4 receptor internalization. Nevertheless, β-arrestin1 can bind and colocalize with cortactin [46], and we cannot exclude their convergent connection.

To go further beyond the Notch3^+high^CXCR4^+high^DP organ-wide dissemination, we investigated their migratory ability in in vivo NSG transplant experiments. Our results highlight that not simply DP, but selected N3^high^DP thymocytes are efficient in positioning into BM, already at day1 post-injection. We report here for the first time how a wave of “thymus-derived” Notch3^+high^CXCR4^+high^DP-cells is able to home and engraft into BM of NSG recipient mice, while the ability of “bone marrow-derived” leukemic cells to infiltrate lymphoid organs and CNS in T-ALL was previously reported [24, 25, 47]. We show that high CXCR4 is per se insufficient, but requires an active Notch stimulus, Notch3 in our model, that possibly potentiates the SDF-1-driven nest of Notch3^+high^CXCR4^+high^DP-cells into BM niches. Our results delineate a new mechanism by which a specific subset of DP T-cells, with a high Notch3 and CXCR4 co-expression and an elevated proliferation rate, are forced to egress from the thymus and fit to rapidly colonize BM and possibly peripheral organs (i.e., the spleen) during T-ALL progression.

In support of our hypothesis, in vivo CXCR4 antagonism by AMD3100 can contrast atypical DP infiltration in the BM of young N3-ICtg mice. Notably, the increased number of N3^high^ and the ratio N3^high^/N3^low^ DP cells infiltrating the spleen further highlight the environmental requirements to sustain Notch3^+high^CXCR4^+high^DP “fitness” in T-cell leukemia propagation. It has been previously shown that primary explants of T-ALL are prone to apoptosis unless stromal stimuli, like Notch ligands (Delta-like1) and SDF-1, are present [25, 48]. In keeping with this we observed a reduced spontaneous apoptosis in SDF-1-treated N3^high^DP-cells, while apoptosis of N3^low^ DP-cells resulted unaffected by SDF-1 treatment. The incompetence of N3^low^DP T-cells to home and engraft into BM and spleen, at day1 and day10 post-injection, underlies the need of a strong Notch signal for committing CXCR4-expressing leukemic cells to propagate and expand in peripheral organs.

In conclusion, our results highlight a new perspective function of CXCR4 combined with Notch3 in selecting and mobilizing “pre-leukemic” DP cells that easily fit to disseminate in early T-ALL progression. Moreover, the Notch3/CXCR4 crosstalk suggests the attractive possibility of combined therapy protocols to precociously target T-ALL cells.

## Materials and methods

### Mice

N3-ICtg mice [18] were bred and maintained as reported in [49]. NOD.Cg-Prkdc^scid^ (NSG) immune-compromised recipient mice (Charles-River Laboratories). Animal experiments performed with at least three animals of each genotype. The number of used mice is reported in each Figure legend. Experimental mice groups were based on age and genotype. No mice were excluded during the experiments. All animal experiments were performed according to Italian D.Lgs. n.26/2014 and European Directive 2010/63/UE.

### Cell culture

Hek293T, TALL-1 cells were maintained as described elsewhere [4, 50] and all are mycoplasma-free.

### Cell transfections and plasmids

Hek293 cells were transiently transfected by TransFectin™Lipid Reagent (BioRad) as in [51]. Expression plasmids used: pCMV-Notch3-IC-HA [18], pcDNA3-βarr1-HA, and pcDNA3-βarr1-S412D-Flag [41] (gift from Robert Lefkowitz; Addgene plasmid #42196).

### siRNA silencing experiments

TALL-1 cells were transfected with 200 nM siRNAs anti-Notch3 (Santa-Cruz, sc-37135) or (Dharmacon, s1623) and their corresponding control scrambled siRNAs (Santa-Cruz, sc-37007 and Dharmacon, D-001810-10-20) using Neon transfection System (Invitrogen) following manufacturer’s recommendations. Ninety-six hours post-transfection, TALL-1 cells were stained with anti-CXCR4 (BD-Biosciences, 560936) or IgM isotype (eBioscience, 17-4341), and analyzed in FACSCalibur with CellQuest software (BD-Biosciences).

### RT-PCR**/**qRT-PCR

Total RNA extraction and reverse transcription-polymerase chain reaction (RT-PCR) were described [52]. CXCR4 and HPRT mRNA levels were determined by TaqMan quantitative real-time RT-PCR (qRT-PCR) performed on cDNA following manufacturer’s instructions (Applied-Biosystems). Data analysis by ΔΔCt method (HPRT use for normalization of mRNA levels).

### Migration assay

In vitro SDF-1-induced (Peprotech) migration was performed with thymocytes (20 and 200 ng/ml) or TALL-1 cells (5 and 50 ng/ml) as described in [53]. An unrelated ligand, SLC (1 μg/ml; Protech) was used. Ninety minutes post-induction, cell-migration was counted by FACS as in [54].

### Protein extracts and immunoblotting

Nuclear-cytosol fractionations of purified DP/CD8^+^ cells, with CD8a(Ly-2) microbeads (Miltenyi-Biotec) following manufacturer’s protocol, and of TALL-1 cells were performed as described [23, 55].

WCE and immunoblotting assays were performed as described [23]. Used antibodies: Anti-Flag(F3165), anti-β-actin(A5441) (Sigma-Aldrich); anti-Phospho-β-arrestin1-Ser412(2416), anti-Notch3(2889) (Cell-Signaling); anti-β-arrestin-1(sc-9182), anti-Phospho-Erk(sc-7383), anti-Erk-1/2(sc-93/sc-154), anti-α-tubulin(sc-803) and anti-LaminB(sc6217) (Santa-Cruz); Anti-CXCR4 (ABCAM; 2074).

### Flow cytometry

T-cells from thymi, blood, and BM were processed as described [22] and stained with anti-CD4-PerCP-Cy5.5, anti-CD8-APC, IgG2bPE(12-4031) (BD-Biosciences); anti-CXCR4-PE (eBioscience); anti-Notch3-Fitch(AF1308) and normal goat-IgG(AB-108-C) (R&D Systems) [53]. Samples analysis on FACSCalibur using CellQuest-software (BD-Biosciences).

CD8-H7(560182) was used with anti-AnnexinV-APC(550474) (BD-Biosciences). FACSCantoII (Becton-Dickinson) analysis with DivaVersion6.1.3 or FlowJoVersion8.5.2 software was performed. Blinded-conducted analysis performed.

### Cell sorting for in vivo cell transfer experiment

Thymocytes of 8-week-old N3-ICtg mice were stained with antibodies abovementioned in flow cytometry.

For in vivo cell transfer experiment, CD4^+^CD8^+^Notch3^+high^CXCR4^+high^ (N3^high^) and CD4^+^CD8^+^Notch3^+low^CXCR4^+high^ (N3^low^) thymocytes of N3-ICtg mice were isolated (purity level > 90%) by FACSAria cell-sorter (BD Biosciences) [56].

2.5 × 10^6^ purified (N3^+high^) or (N3^+low^) DP T-cells were i.v. injected in NSG mice.

At day1 and day10 post-injection, 2 × 10^6^ cells from spleen and BM of NSG recipient mice were stained as above-mentioned in flow cytometry. FACSCantoII (Becton-Dickinson) analysis with DivaVersion6.1.3 or FlowJoVersion8.5.2 software was performed.

### In vivo administration of AMD3100

Three- to four-week-old N3-ICtg mice were IP injected daily with AMD3100 (10 mg/kg; Sigma-Aldrich) or vehicle (PBS). FACS analysis of BM cells was performed at day1 and day10. Antibodies are mentioned above in flow cytometry [53].

### Statistical analysis

Comparison of two groups using unpaired *t*-test or Mann–Whitney test was done. Comparison of more groups using one-way ANOVA (Tukey’s post-test and Kruskal–Wallis test) was done. A *p*-value of <0.05 was considered as statistically significant (*p < 0.05, **p< 0.01, ***p < 0.001, ****P < 0.0001; ^§§^*p* < 0.01; ^§§§§^*p* < 0.0001). At least three independent experiments are reported as mean ± SD (the repeat number was increased according to effect size or sample variation). Estimation of sample’s size considering variation and mean of samples was done. No statistical method was used to predetermine sample size. No animals/samples were excluded from the analysis. Statistical analysis was conducted with PRISM-program (GraphPad).

### In vivo BrdU labeling and Ki67 staining

Each 8-week-old N3-ICtg mouse was IP injected with 1.5 mg/150 ml BrdU (Sigma-Aldrich; B9285) in PBS; 24 h post-IP-injection, anti-CD4/anti-CD8/anti-Notch3 extracellular staining was followed either by anti-BrdU (BioLegend; 339807) [57] or fixed and permeabilized for intracellular antigen detection of BV510mouse Aati-Ki-67(BD-563462).

### In silico analysis of T-ALL patients’ deposited data

Blood samples from 117 pediatric T-ALL patients [42] were selected and analyzed for correlation between CXCR4 and ARRB1. Expression probe sets 217028_at representing CXCR4 and 222912_at representing ARRB1 were used.

## Electronic supplementary material

Supplemental Material

## References

[1] Weng AP, Ferrando AA, Lee W, Morris JP, Silverman LB, Sanchez-Irizarry C (2004). Activating mutations of NOTCH1 in human T cell acute lymphoblastic leukemia. Science.

[2] Bellavia D, Campese AF, Checquolo S, Balestri A, Biondi A, Cazzaniga G (2002). Combined expression of pTα and Notch3 in T cell leukemia identifies the requirement of preTCR for leukemogenesis. Proc Natl Acad Sci USA.

[3] CAMPESE A, BELLAVIA D, GULINO A, SCREPANTI I (2003). Notch signalling at the crossroads of T cell development and leukemogenesis. Seminars in Cell & Developmental Biology.

[4] Bernasconi-Elias P, Hu T, Jenkins D, Firestone B, Gans S, Kurth E (2016). Characterization of activating mutations of NOTCH3 in T cell acute lymphoblastic leukemia and anti-leukemic activity of NOTCH3 inhibitory antibodies. Oncogene.

[5] Washburn T, Schweighoffer E, Gridley T, Chang D, Fowlkes B, Cado D (1997). Notch activity influences the αβ versus γδ T cell lineage decision. Cell.

[6] Felli MP, Maroder M, Mitsiadis TA, Campese AF, Bellavia D, Vacca A (1999). Expression pattern of Notch1, 2 and 3 and Jagged1 and 2 in lymphoid and stromal thymus components: distinct ligand–receptor interactions in intrathymic T cell development. Int Immunol.

[7] Martins VC, Busch K, Juraeva D, Blum C, Ludwig C, Rasche V (2014). Cell competition is a tumour suppressor mechanism in the thymus. Nature.

[8] Chatterjee S, Azad BB, Nimmagadda S (2014). The intricate role of CXCR4 in cancer. Adv Cancer Res.

[9] Petrie HT, Zúñiga-Pflücker JC (2007). Zoned out: functional mapping of stromal signaling microenvironments in the thymus. Annu Rev Immunol.

[10] Poznansky MC, Olszak IT, Foxall R, Evans RH, Luster AD, Scadden DT (2000). Active movement of T cells away from a chemokine. Nat Med.

[11] Cyster JG (2002). Chemorepulsion and thymocyte emigration. J Clin Investig.

[12] Ara T, Itoi M, Kawabata K, Egawa T, Tokoyoda K, Sugiyama T (2003). A role of CXC chemokine ligand 12/stromal cell-derived factor-1/pre-B cell growth stimulating factor and its receptor CXCR4 in fetal and adult T cell development in vivo. J Immunol.

[13] Plotkin J, Prockop SE, Lepique A, Petrie HT (2003). Critical role for CXCR4 signaling in progenitor localization and T cell differentiation in the postnatal thymus. J Immunol.

[14] Janas ML, Varano G, Gudmundsson K, Noda M, Nagasawa T, Turner M (2010). Thymic development beyond β-selection requires phosphatidylinositol 3-kinase activation by CXCR4. J Exp Med.

[15] Trampont PC, Tosello-Trampont AC, Shen Y, Duley AK, Sutherland AE, Bender TP (2010). CXCR4 acts as a costimulator during thymic [beta]-selection. Nat Immunol.

[16] Radtke F, MacDonald HR, Tacchini-Cottier F (2013). Regulation of innate and adaptive immunity by Notch. Nat Rev Immunol.

[17] Janas ML, Turner M (2010). Stromal cell-derived factor 1α and CXCR4: newly defined requirements for efficient thymic β-selection. Trends Immunol.

[18] Bellavia D, Campese AF, Alesse E, Vacca A, Felli MP, Balestri A (2000). Constitutive activation of NF‐κB and T‐cell leukemia/lymphoma in Notch3 transgenic mice. EMBO J.

[19] Bellavia D, Mecarozzi M, Campese AF, Grazioli P, Talora C, Frati L (2007). Notch3 and the Notch3‐upregulated RNA‐binding protein HuD regulate Ikaros alternative splicing. EMBO J.

[20] Pear WS, Aster JC, Scott ML, Hasserjian RP, Soffer B, Sklar J (1996). Exclusive development of T cell neoplasms in mice transplanted with bone marrow expressing activated Notch alleles. J Exp Med.

[21] Franciosa G, Diluvio G, Del Gaudio F, Giuli M, Palermo R, Grazioli P (2016). Prolyl-isomerase Pin1 controls Notch3 protein expression and regulates T-ALL progression. Oncogene.

[22] Felli MP, Vacca A, Calce A, Bellavia D, Campese AF, Grillo R (2005). PKC [theta] mediates pre-TCR signaling and contributes to Notch3-induced T-cell leukemia. Oncogene.

[23] Vacca A, Felli MP, Palermo R, Di Mario G, Calce A, Di Giovine M (2006). Notch3 and pre‐TCR interaction unveils distinct NF‐κB pathways in T‐cell development and leukemia. EMBO J.

[24] Passaro D, Irigoyen M, Catherinet C, Gachet S, De Jesus CDC, Lasgi C (2015). CXCR4 is required for leukemia-initiating cell activity in T cell acute lymphoblastic leukemia. Cancer Cell.

[25] Pitt LA, Tikhonova AN, Hu H, Trimarchi T, King B, Gong Y (2015). CXCL12-producing vascular endothelial niches control acute T cell leukemia maintenance. Cancer Cell.

[26] Halkias J, Melichar HJ, Taylor KT, Ross JO, Yen B, Cooper SB (2013). Opposing chemokine gradients control human thymocyte migration in situ. J Clin Investig.

[27] Campese AF, Garbe AI, Zhang F, Grassi F, Screpanti I, Von Boehmer H (2006). Notch1-dependent lymphomagenesis is assisted by but does not essentially require pre-TCR signaling. Blood.

[28] Kuksin CA, Gonzalez-Perez G, Minter LM (2015). CXCR4 expression on pathogenic T cells facilitates their bone marrow infiltration in a mouse model of aplastic anemia. Blood.

[29] Geminder H, Sagi-Assif O, Goldberg L, Meshel T, Rechavi G, Witz IP (2001). A possible role for CXCR4 and its ligand, the CXC chemokine stromal cell-derived factor-1, in the development of bone marrow metastases in neuroblastoma. J Immunol.

[30] Ben-Baruch A (2009). Site-specific metastasis formation: chemokines as regulators of tumor cell adhesion, motility and invasion. Cell Adh Migr.

[31] Russell HV, Hicks J, Okcu MF, Nuchtern JG (2004). CXCR4 expression in neuroblastoma primary tumors is associated with clinical presentation of bone and bone marrow metastases. J Pediatr Surg.

[32] Scupoli MT, Donadelli M, Cioffi F, Rossi M, Perbellini O, Malpeli G (2008). Bone marrow stromal cells and the upregulation of interleukin-8 production in human T-cell acute lymphoblastic leukemia through the CXCL12/CXCR4 axis and the NF-κB and JNK/AP-1 pathways. Haematologica.

[33] Uckun FM, Sensel MG, Sun L, Steinherz PG, Trigg ME, Heerema NA (1998). Biology and treatment of childhood T-lineage acute lymphoblastic leukemia. Blood.

[34] Liu T, Li X, You S, Bhuyan SS, Dong L (2015). Effectiveness of AMD3100 in treatment of leukemia and solid tumors: from original discovery to use in current clinical practice. Exp Hematol Oncol.

[35] Mirandola L, Chiriva‐Internati M, Montagna D, Locatelli F, Zecca M, Ranzani M (2012). Notch1 regulates chemotaxis and proliferation by controlling the CC‐chemokine receptors 5 and 9 in T cell acute lymphoblastic leukaemia. J Pathol.

[36] Marchese A, Trejo J (2013). Ubiquitin-dependent regulation of G protein-coupled receptor trafficking and signaling. Cell Signal.

[37] Puca L, Brou C (2014). α-Arrestins–new players in Notch and GPCR signaling pathways in mammals. J Cell Sci.

[38] Lin FT, Miller WE, Luttrell LM, Lefkowitz RJ (1999). Feedback regulation of β-arrestin1 function by extracellular signal-regulated kinases. J Biol Chem.

[39] Talora C, Campese AF, Bellavia D, Pascucci M, Checquolo S, Groppioni M (2003). Pre‐TCR‐triggered ERK signalling‐dependent downregulation of E2A activity in Notch3‐induced T‐cell lymphoma. EMBO Rep.

[40] Talora C, Cialfi S, Oliviero C, Palermo R, Pascucci M, Frati L (2006). Cross talk among Notch3, pre-TCR, and Tal1 in T-cell development and leukemogenesis. Blood.

[41] Luttrell L, Ferguson S, Daaka Y, Miller W, Maudsley S, Della Rocca G (1999). β-Arrestin-dependent formation of β2 adrenergic receptor-Src protein kinase complexes. Science.

[42] Homminga I, Pieters R, Langerak AW, de Rooi JJ, Stubbs A, Verstegen M (2011). Integrated transcript and genome analyses reveal NKX2-1 and MEF2C as potential oncogenes in T cell acute lymphoblastic leukemia. Cancer Cell.

[43] Van Vlierberghe P, Ferrando A (2012). The molecular basis of T cell acute lymphoblastic leukemia. J Clin Investig.

[44] Lin FT, Krueger KM, Kendall HE, Daaka Y, Fredericks ZL, Pitcher JA (1997). Clathrin-mediated endocytosis of the β-adrenergic receptor is regulated by phosphorylation/dephosphorylation of β-arrestin1. J Biol Chem.

[45] Hupfeld CJ, Resnik JL, Ugi S, Olefsky JM (2005). Insulin-induced β-arrestin1 Ser-412 phosphorylation is a mechanism for desensitization of ERK activation by Gαi-coupled receptors. J Biol Chem.

[46] Goertzen CG, Dragan M, Turley E, Babwah AV, Bhattacharya M (2016). KISS1R signaling promotes invadopodia formation in human breast cancer cell via β-arrestin2/ERK. Cell Signal.

[47] Jost TR, Borga C, Radaelli E, Romagnani A, Perruzza L, Omodho L (2016). Role of CXCR4-mediated bone marrow colonization in CNS infiltration by T cell acute lymphoblastic leukemia. J Leukoc Biol.

[48] Armstrong F, de la Grange PB, Gerby B, Rouyez MC, Calvo J, Fontenay M (2009). NOTCH is a key regulator of human T-cell acute leukemia initiating cell activity. Blood.

[49] Barbarulo A, Grazioli P, Campese AF, Bellavia D, Di Mario G, Pelullo M (2011). Notch3 and canonical NF-κB signaling pathways cooperatively regulate Foxp3 transcription. J Immunol.

[50] Pelullo M, Quaranta R, Talora C, Checquolo S, Cialfi S, Felli MP (2014). Notch3/Jagged1 circuitry reinforces notch signaling and sustains T-ALL. Neoplasia.

[51] Massimi I, Ciuffetta A, Temperilli F, Ferrandino F, Zicari A, Pulcinelli FM (2015). Multidrug resistance protein-4 influences aspirin toxicity in human cell line.. Mediators Inflamm.

[52] Quaranta R, Pelullo M, Zema S, Nardozza F, Checquolo S, Lauer DM (2017). Maml1 acts cooperatively with Gli proteins to regulate sonic hedgehog signaling pathway. Cell Death Dis.

[53] Bernardini G, Sciume G, Bosisio D, Morrone S, Sozzani S, Santoni A (2008). CCL3 and CXCL12 regulate trafficking of mouse bone marrow NK cell subsets. Blood.

[54] Bernardini G, Kim JY, Gismondi A, Butcher EC, Santoni A (2005). Chemoattractant induces LFA-1 associated PI 3K activity and cell migration that are dependent on Fyn signaling. FASEB J.

[55] Checquolo S, Palermo R, Cialfi S, Ferrara G, Oliviero C, Talora C (2010). Differential subcellular localization regulates c-Cbl E3 ligase activity upon Notch3 protein in T-cell leukemia. Oncogene.

[56] Campese AF, Grazioli P, de Cesaris P, Riccioli A, Bellavia D, Pelullo M (2014). Mouse Sertoli cells sustain de novo generation of regulatory T cells by triggering the notch pathway through soluble JAGGED1. Biol Reprod.

[57] Parretta E, Cassese G, Santoni A, Guardiola J, Vecchio A, Di Rosa F (2008). Kinetics of in vivo proliferation and death of memory and naive CD8 T cells: parameter estimation based on 5-bromo-2′-deoxyuridine incorporation in spleen, lymph nodes, and bone marrow. J Immunol.

