# Supplementary material for: Intrathymic Notch3 and CXCR4 combinatorial interplay facilitates T-cell leukemia propagation
Source: Oncogene. 2018 Jul 23;37(49):6285–98. doi: 10.1038/s41388-018-0401-2 (PMC6284016; doi:10.1038/s41388-018-0401-2)
Supplement: Supplementary file 1 — Supplemental Material [file 41388_2018_401_MOESM1_ESM.pdf]

# Intrathymic Notch3 and CXCR4 combinatorial interplay facilitates T-cell leukemia propagation.

Ferrandino F.[1], Bernardini G.[2,3], Tsaouli G.[2,3], Grazioli P.[1], Campese A.F.[2], Noce C.[2], Ciuffetta A.[2], Vacca A.[1], Besharat ZM.[1], Bellavia D.[2], Screpanti I.[2,4,5,], and Felli M.P.[1,4,5].

**Supplementary Information:**

Supplementary Figures S1-9

## Supplementary Figures

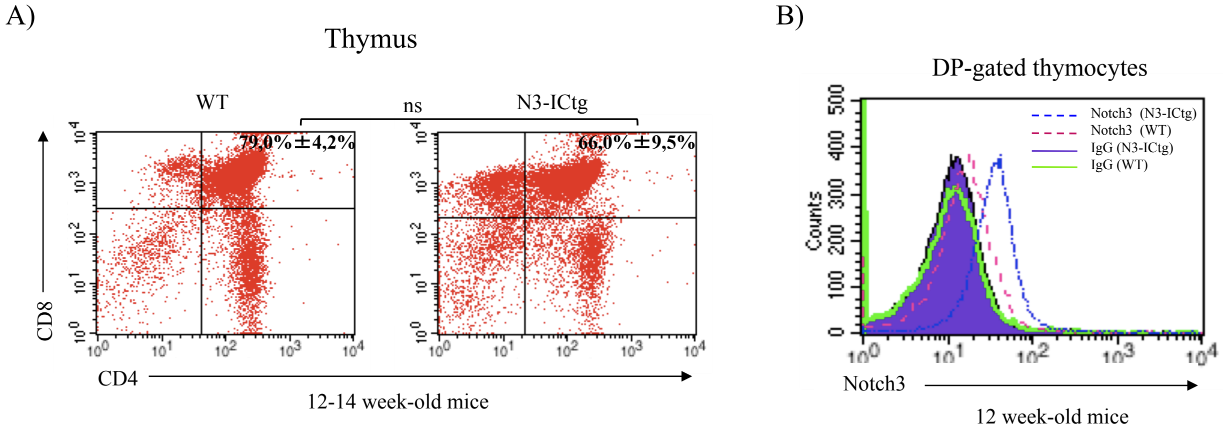


**Supplementary Figure S1. Notch3 expression lasting in CD4$^+$CD8$^+$ thymocytes of 12-14 week-old mice.** A) FACS analysis of CD4$^+$CD8$^+$ thymocytes distribution in representative WT and Notch3-IC transgenic (N3-ICtg) thymi. Average percentages ± SD of DP thymocytes are indicated in plots. (not significant, ns; Student's t-test). B) FACS analysis of Notch3 cell-surface expression of DP-gated thymocytes of WT (Red dotted line) and N3-ICtg (blue dotted line) of 12 week-old mice. Control isotype is shown as green (WT) and purple (N3-ICtg) line.

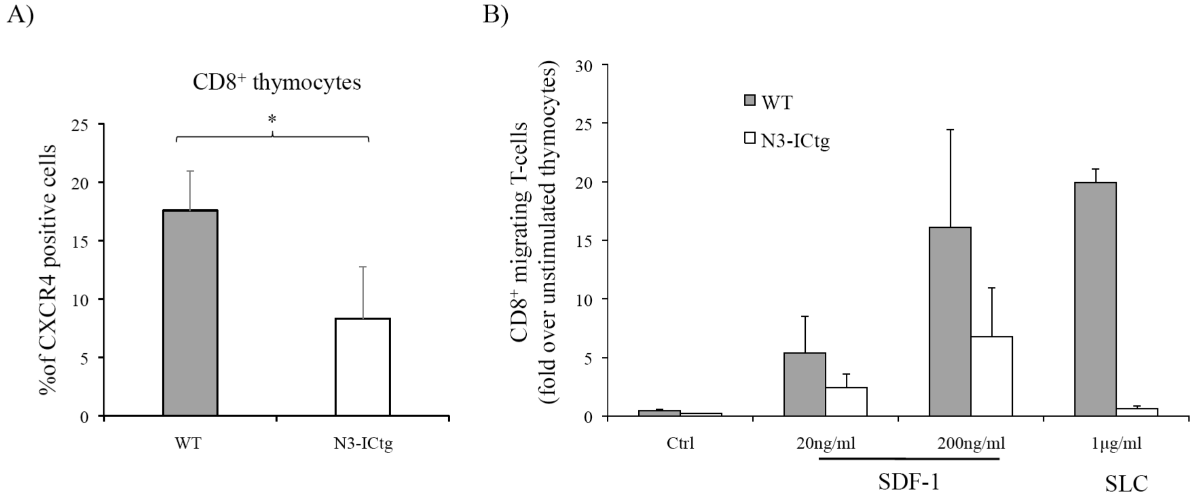


**Supplementary Figure S2. Downmodulation of CXCR4 cell-surface expression and migratory ability of CD8$^+$ thymocytes in N3-ICtg mice.** In A) the percentages of CD8$^+$CXCR4$^+$ positive T-cells by comparing N3-ICtg versus WT thymocytes. B) *In vitro* migration of CD8$^+$ thymocytes of N3-ICtg and WT mice in response to 20ng/ml and 200ng/ml of CXCL12/SDF-1 or to 1 µg/ml of an unrelated ligand (Secondary Lymphoid-tissue Chemokine, SLC) in a transwell chemotaxis assay (90 minutes). Evaluation was performed as fold increase of CD8$^+$ migrating thymocytes percentages in response to SDF-1 stimulated versus unstimulated thymocytes. P-values were calculated using a Student's t-test (*p<0.05).

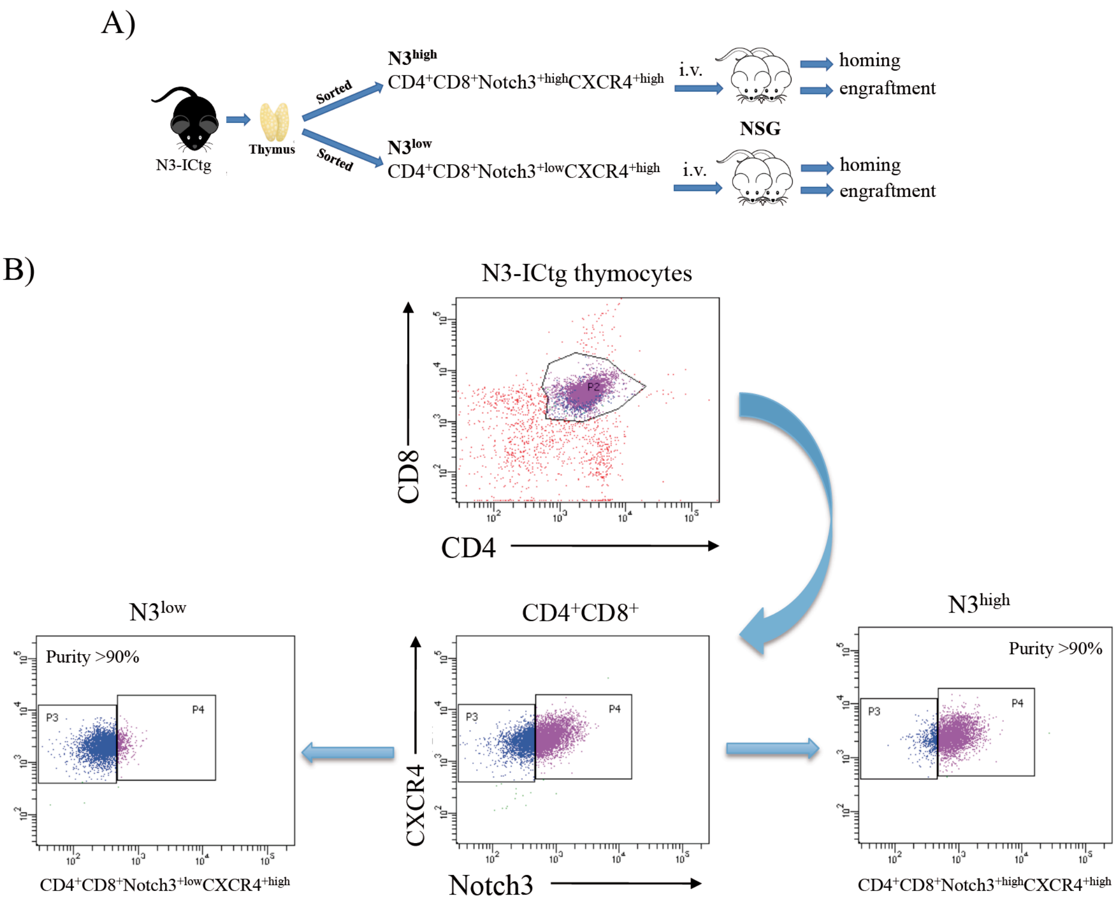


**Supplementary Figure S3. *In vivo* cell transfer in NSG recipient mice. The sorting strategy of N3$^{high}$ and N3$^{low}$ of N3-ICtg DP thymocytes.** A) Schematic representation of the experiment of thymocytes transfer in NSG mice. Sorted CD4$^+$CD8$^+$Notch3$^{+high}$CXCR4$^{+high}$ (N3$^{high}$) or CD4$^+$CD8$^+$Notch3$^{+low}$CXCR4$^{+high}$ (N3$^{low}$) thymocytes were intravenously (i.v.) injected in NSG mice. Post-injection flow-cytometry analyses of bone marrow and spleen cells were performed after 1day for homing and after 10 days for engraftment evaluation. B) Schematized in the figure is the flow-cytometry experimental procedure used to purify CD4$^+$CD8$^+$Notch3$^{+high}$CXCR4$^{+high}$ (N3$^{high}$) and CD4$^+$CD8$^+$Notch3$^{+low}$CXCR4$^{+high}$ (N3$^{low}$) thymocytes from 8 week-old N3-ICtg mice. For FACSAria sorting, a four color staining was performed. The purity of the selected T-cells is > 90%. N3$^{high}$ and/or N3$^{low}$ thymocytes were separately injected intravenously (2,5 x10$^6$ purified T-cells i.v.) in immunocompromized NSG mice.

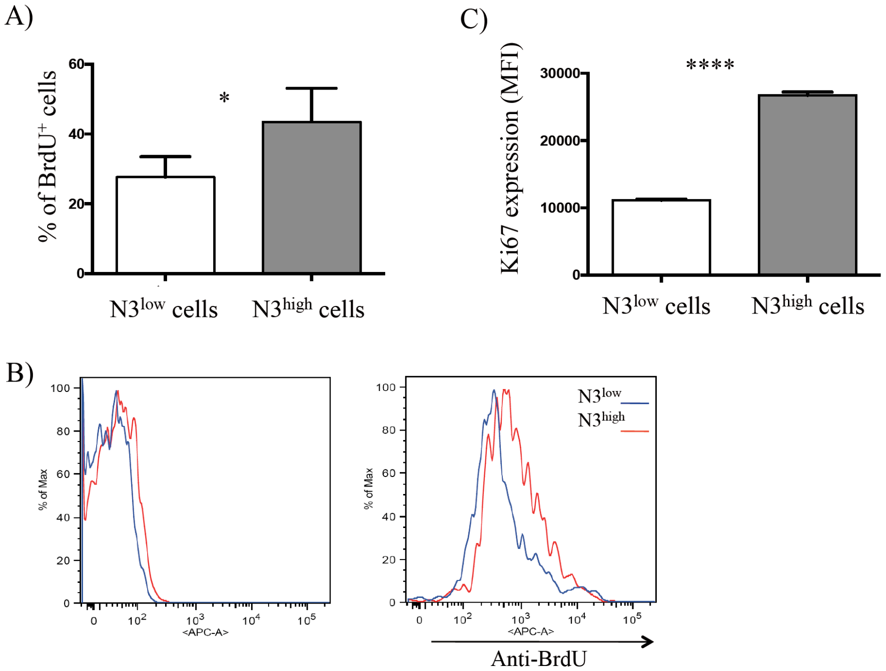


**Supplementary Figure S4. Analysis of *in vivo* and *ex vivo* proliferation rate of N3$^{high}$ and N3$^{low}$ DP thymocytes of N3-ICtg.** A) *In vivo* BrdU labeling of DP Notch3$^{+}$ thymocytes of 8 weeks-old N3-ICtg mice. 24h post-injection the percentage of BrdU positive cells was evaluated at FACSCanto II. P-values were calculated using a Student's t-test (*p<0.05). B) Flow cytometry analysis with anti-BrdU of Notch3$^{high}$ (red line) as opposed to Notch3$^{low}$ thymocytes (blue line). C) *Ex vivo* freshly isolated thymocytes of 8 weeks-old N3-ICtg mice were fixed and permeabilized for Ki67 intracellular staining. The Mean Fluorescence Intensity (MFI) is reported. P-values were calculated using a Student's t-test (****p<0.0001).

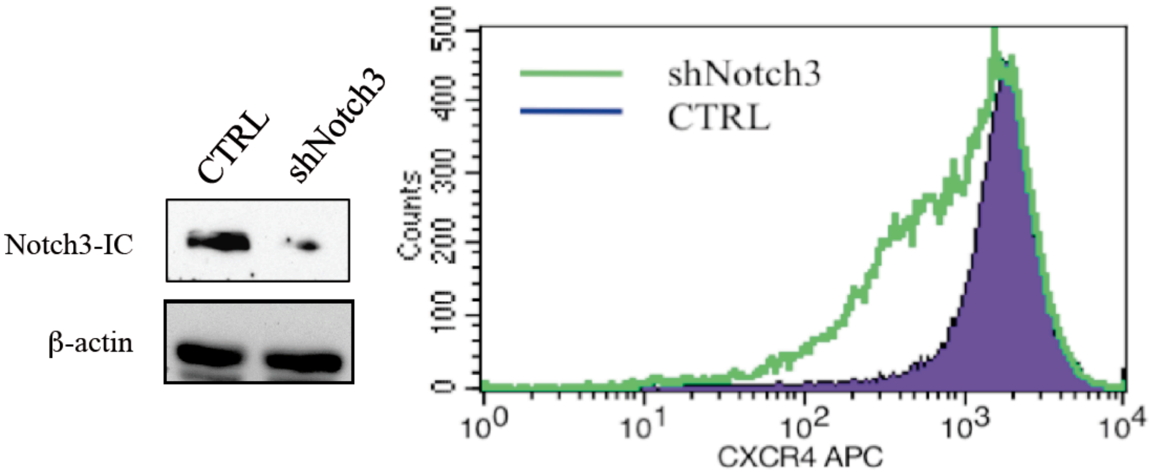


**Supplementary Figure S5.** Left panel, Western blot analysis to monitor Notch3 silencing and β-actin expression. Right panel, FACS analysis of TALL-1 cells silenced with a second independent Notch3 hairpin produced by Dharmacon. 96h post-silencing, CXCR4 expression was monitored in Control (CTRL) versus Notch3 silenced (shNotch3) TALL-1 cells.

A)

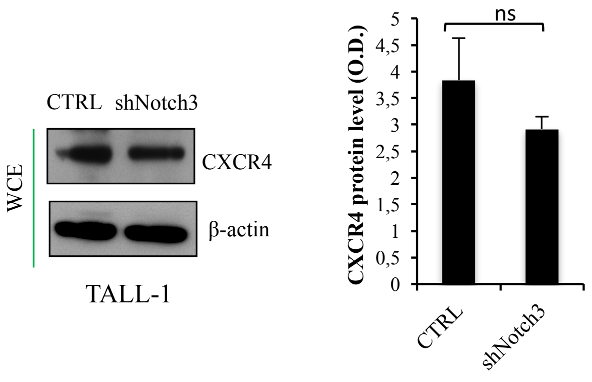


B)

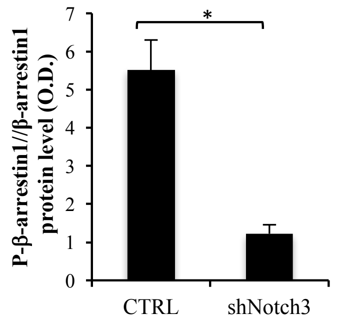


C)

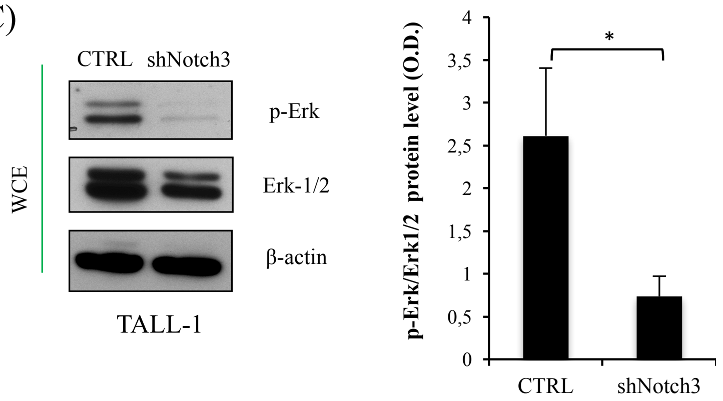


**Supplementary Figure S6. Notch3 silencing impairs phosphorylation of Erk and β-arrestin1 in the human TALL-1 cells.** Whole cell extract (WCE) derived from Notch3-silenced (SantaCruz assay) TALL-1 (shNotch3) versus control cells (CTRL), A) were probed with antibodies for CXCR4 and β-actin. Right panel, Optical Densitometry (OD) of CXCR4 protein analyzed in all the experiments performed. B) Quantification of the ratio between phospho-β-arrestin1 over the total β-arrestin1 levels (p-β-arrestin1/β-arrestin1) in CTRL and shNotch3 TALL-1 cells. C) Western blot analysis of protein extract of shNotch3 and CTRL cells were immunoblotted for Erk1/2 phosphorylation (p-Erk) or for total Erk protein (Erk1/2) and β-actin. Right panel, Optical Densitometry (OD) of the ratio between p-Erk normalized to the total Erk1/2 signal (p-Erk/Erk1/2) analyzed in all the experiments performed. Average fold change of each protein was calculated by normalization to β-actin levels. P-values, calculated using Student's t-test (ns, not significant; *P<0.05). Densitometry was performed on scanned immunoblot images using ImageJ.

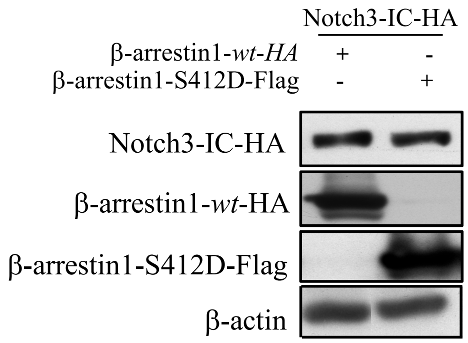


**Supplementary Figure S7. Hek293 cells were transiently co-transfected with N3-IC and β-arrestin1-*wt* or mutant β-arrestin1-S412D protein.** 1.4 μg of *wt* (β-arrestin1-*wt*-HA) or 1.4 μg of the mutant S412D (β-arrestin1-S412D-Flag), a β-arrestin1 locked in the phosphorylated from, were transfected in Hek293 cells. All the samples were co-transfected with an active intracellular form of Notch3 (Notch3-IC-HA) (0.7 μg). Average fold change of each protein was calculated by normalization to β-actin levels.

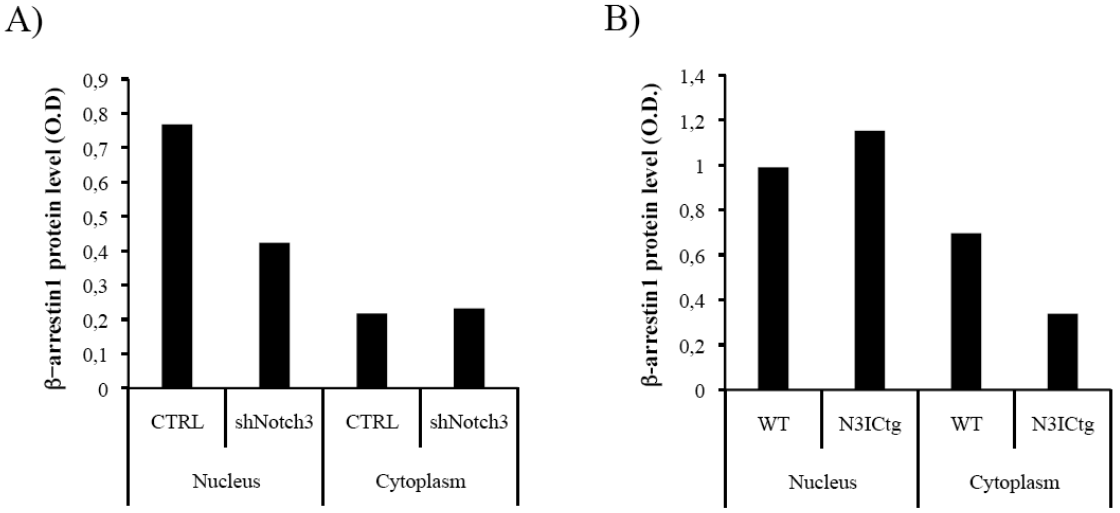


**Supplementary Figure S8. Differential β-arrestin1 protein levels in nucleus and cytoplasm.** Optical densitometry (OD) of β-arrestin1 in fractionated extract A) of Notch3 silenced TALL-1 (shNotch3) and control (CTRL) cells, and B) of WT and N3-ICtg DP/$CD8^+$ purified thymocytes of 6-8 week-old mice. Average fold change of each protein was calculated by normalization to lamin-B or to α-tubulin levels. Densitometry was performed on scanned immunoblot images using ImageJ.

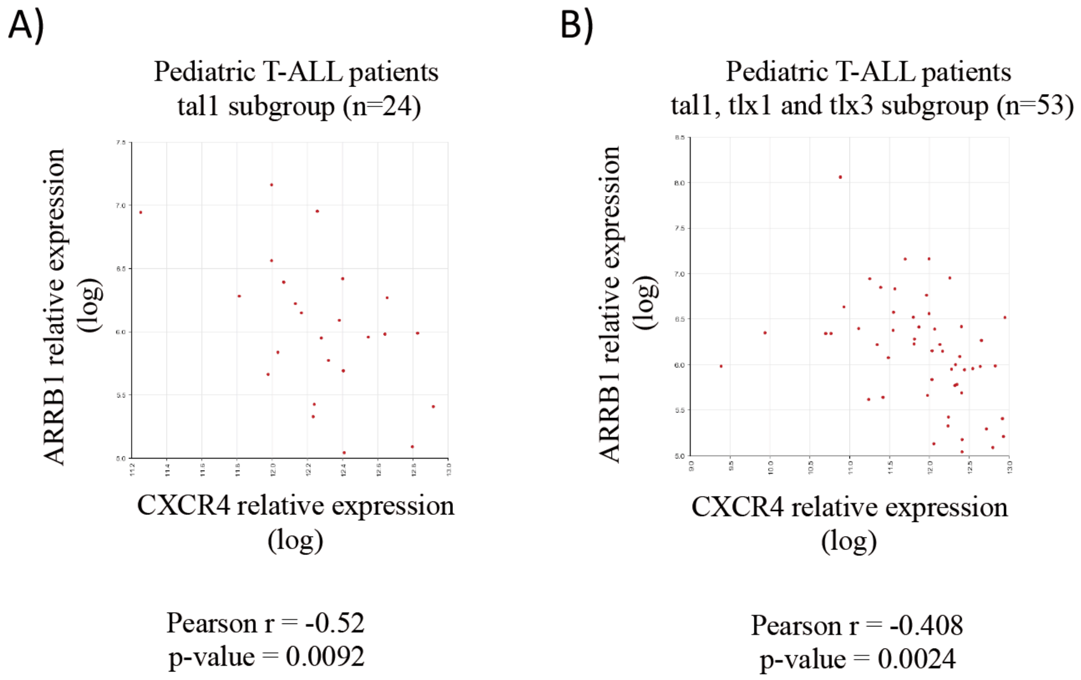


**Supplementary Figure S9. CXCR4 and ARRB1 levels correlate in T-ALL patients.**
Graphs showing the inverse correlation between CXCR4 (CXCR4) and β-arrestin1 (ARRB1) gene expression levels obtained by an *in silico* analysis using the expression of probes set 217028_at and set 222912_at representing the CXCR4 and ARRB1, respectively, in a cohort of (A) 24 tal1 pediatric T-ALL patients (B) 53 (tal1, tlx1 and tlx3 subgroup) pediatric T-ALL patients. In all graphs (A-B), each dot corresponds to one patient and the expression value of CXCR4 and ARRB1 is given in log2 scale after normalizing data with mas5.0 normalization algorithm. The X-Y axis represent CXCR4 and ARRB1 expression levels, respectively. The index Pearson r expresses the linear relation between paired samples and p-values were calculated using Student's t-test.
